# Comparison of cell state models derived from single-cell RNA sequencing data: graph versus multi-dimensional space

**DOI:** 10.3934/mbe.2022395

**Published:** 2022-06-10

**Authors:** Heyrim Cho, Ya-Huei Kuo, Russell C. Rockne

**Affiliations:** 1 Department of Mathematics, University of California Riverside, Riverside, CA, USA; 2 Department of Hematologic Malignancies Translational Science, City of Hope, Duarte, CA, USA; 3 Department of Computational and Quantitative Medicine, Division of Mathematical Oncology, City of Hope, Duarte, CA, USA; 4 Interdisciplinary Center for Quantitative Modeling in Biology, University of California Riverside, Riverside, CA, USA

**Keywords:** next generation sequencing data, cell state evolution, phenotype structured models, partial differential equation, hematopoeisis

## Abstract

Single-cell sequencing technologies have revolutionized molecular and cellular biology and stimulated the development of computational tools to analyze the data generated from these technology platforms. However, despite the recent explosion of computational analysis tools, relatively few mathematical models have been developed to utilize these data. Here we compare and contrast two cell state geometries for building mathematical models of cell state-transitions with single-cell RNA-sequencing data with hematopoeisis as a model system; (i) by using partial differential equations on a graph representing intermediate cell states between known cell types, and (ii) by using the equations on a multi-dimensional continuous cell state-space. As an application of our approach, we demonstrate how the calibrated models may be used to mathematically perturb normal hematopoeisis to simulate, predict, and study the emergence of novel cell states during the pathogenesis of acute myeloid leukemia. We particularly focus on comparing the strength and weakness of the graph model and multi-dimensional model.

## 1. Introduction

The ability to apply genome sequencing methods to single-cells has revolutionized biology [1]. Technologies enabling single-cell sequencing are advancing rapidly, with datasets as large as hundreds of thousands of cells are common [2]. RNA-sequencing is currently the most prevalent form of single-cell genomic analysis [1]. The sequencing of RNA at the cellular level enables the interrogation of gene transcription, which is used as a high-dimensional fingerprint which characterizes the identity of the cell. For this reason, single-cell RNA-sequencing data (scRNA-seq) has been used as a tool to study cell identity and state-transitions at the cellular level.

The most frequently studied cell state-transition is cellular differentiation; the process of a cell and its progeny to perform specialized tasks through transformation from a less differentiated stem-like state to a more differentiated state. ScRNA-seq is used to identify cells in various states of differentiation primarily through one or both of two primary methods: 1) clustering of cells with similar features [3], or 2) though trajectory inference (TI) [4]. Clustering analysis relies on the definition of a similarity metric, and may rely on a pre-defined number of clusters (e.g., k-means), or may use optimization methods to identify clusters (e.g., Leiden). There are a wide variety of clustering methods and similarity metrics to choose from, which may give drastically different results [5]. Similarly, trajectory inference methods may use pre-defined relationships between cells or may use optimization methods to identify these relationships to construct graphs which are then used to infer paths, or trajectories, between cell states. In addition, various approaches aim to characterize the cell fate landscape, for instance, by a parameterized landscape based on bifurcation analysis [6, 7], by using a measure of entropy of cell states: SCENT [8] and scEpath [9], or by mapping cells to a landscape on optimized parameters: HopLand [10] and Topslam [11].

A significant limitation of these approaches is if the graph structure and underlying relationships between the cells is unknown. As shown in a comprehensive review of trajectory inference methods by Saelens et al. (2019) [4], most TI algorithms have difficulty inferring even simple graphs which may include cycles or disconnected subgraphs. Because of the limitations of clustering and trajectory inference in analysing these data, we suggest that a hypothesis-driven and mathematical approach to the analysis of scRNA-seq data to study cell state transitions is warranted.

Moreover, single-cell genomic sequencing suffers from a number of challenges in analysis. Beyond the several choices to be made for even simple analyses such as clustering or visualization, the data may be sparse and incomplete. Gene “drop outs” and background signal (noise) can complicate differential expression and clustering analyses. For this reason, analysis of these data have remained fairly superficial despite the wealth of information contained in these high-dimensional datasets. Moreover, results obtained from analysis of single-cell sequencing datasets may be very sensitive to choices in the method of analysis and algorithm parameters. To date, single-cell sequencing data have not been effectively leveraged as inputs into mathematical models.

**Figure 1.**
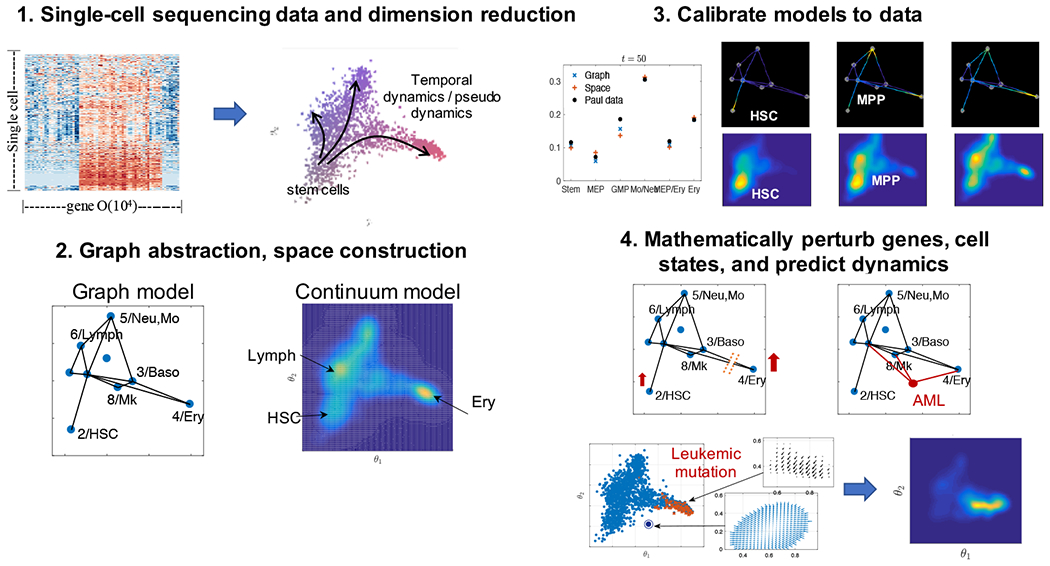
Step-by-step illustration of our modeling process. 1. Processed single-cell sequencing expression matrices are represented in a reduced dimension space through one of many dimension reduction techniques such as diffusion mapping, PCA, t-SNE, or UMAP. 2. Cell clusters are inferred to construct the cell state geometry either the graph or multi-dimensional continuum of cell states. 3. From these representations, models are calibrated to the transport of cell distribution along the graph or in the cell state space. 4. The models can then be utilized to perturb genes and cell states. The calibrated models predict cell state-transitions and the emergence of novel cell states.

Here we compare two cell state geometries of cell state-transitions modeling with scRNA-seq data. Building on our prior work [12], we model cell differentiation as a continuous process. To elaborate this concept, when cell type-A becomes cell type-B, the cell states during the transition process are often classified into more steps as type-A½ or types-A¼, 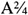, A¾. The continuous cell states can be considered as a limit of these states. We develop phenotype structured cell state models assuming continuous cell states using reaction-diffusion-advection partial differential equations (PDE) solved on: (i) an abstracted graph and (ii) a multi-dimensional continuum space. We compare and contrast these two cell state geometries with hematopoeisis as a model system. This manuscript is structured as follows: first we present the PDE model on a graph and in continuous space, then we apply the model to two datasets, see [13,14]. We examine the impact of various graph construction and trajectory inference methods on the geometry of the cell state space, and solve the model on these geometries. We then use the model the study the effects of perturbing 1) the graph structure 2) expression of select subsets of genes 3) and cell state transition dynamics by perturbing flow on the graph or by modifying the dynamics in the continuous space. We predict novel dynamics of leukemia pathogenesis by perturbing normal hematopoeisis and conclude with a comparative analysis of our approach and description of future directions for mathematical modeling with single-cell genomic sequencing data. A summary of our workflow is shown in Figure 1.

## 2. Materials and method

### 2.1. Modeling cell state-transitions in a continuous cell state-space

In this section we develop PDE models of cell dynamics in the continuous phenotype space identified by dimension reduction techniques. For a given single-cell genomic sequencing dataset

$${\left\{g^{i}\right\}}_{i=1}^{N},\;g^{i}=\left(g_{1}^{i},g_{2}^{i},\ldots ,g_{G}^{i}\right)\in R^{G},$$

where *N* is the number of cells and ***g***^*i*^ is a *G*-dimensional vector of gene expression of the *i*-th cell, the dimension reduction method can be written as an operator $P:R^{G}\to \text{Γ}\subset R^{n}$ where the reduced dimensional space is truncated at the *n*-th dimension and *n* ≪ *G*. We denote the reduced space variable as

(2.1) $$\theta =P(g)\in \text{Γ}\subset R^{n},\;\theta =\left(\theta_{1},\theta_{2},\ldots ,\theta_{n}\right),$$

and the *i*-th single-cell data can be transformed into the reduced space as $P\left(g^{i}\right)=\theta^{i}=\left(\theta_{1}^{i},\theta_{2}^{i},\ldots ,\theta_{n}^{i}\right)$. Various dimension reduction techniques exist to construct a mapping P, including principal component analysis (PCA), diffusion mapping, and t-distributed stochastic neighbor embedding (t-SNE). While different techniques provide different shapes and differentiation spaces, we choose diffusion mapping due to its ability to capture non-linear structure of high-dimensional data, and to well reproduce global trajectory of data [15]. We comment that if the reduced dimensional space is not clear to truncate at a low-dimension, one can consider low-dimensional marker genes according to the cell state of interest, and semi-supervised learning approach can be applied to obtain the low-dimensional reduced space. We also comment that it is common to remove the effect of cell cycles from the gene expression data to eliminate the cell state regarding their location along the cell cycle [16].

### 2.2. PDE model of cell state-transitions on a multi-dimensional reduced component space

We first develop a cell state model that describes the dynamics of cell distribution *u*(*t, θ*) on the reduced component space Γ, where *θ* ∈ Γ is the variable that represents continuous cell state. Three highly distinctive dynamic regimes of the cell states are considered, namely, directed cell transition, birth-death process, and random phenotypic instability. Such model can be written as an advection-reaction-diffusion PDE that governs the cell distribution *u*(*t, θ*) as

(2.2) $$\partial_{t}u(t,\theta )=-\nabla \cdot (V(t,\theta )u(t,\theta ))+R(\theta ,u(t,\theta ))+\nabla \cdot (D(\theta )\nabla u(t,\theta )),$$

with zero Dirichlet boundary condition. The three terms in our equation that involve parameterized functions *V*, *R*, and *D*, represent cell differentiation, population growth, and phenotypic instability, respectively.

Let us first describe the advection term $V\in R^{n}$ that represents directed cell differentiation, where we propose two candidates for modeling *V*, denoted as **v**_1_ and **v**_2_. The first candidate **v**_1_ assumes an attractor cell states of homeostasis. Assuming that the magnitude of phenotypic instability is with a magnitude *v*, that is, *D*(*θ*) = *ν*, one can compute the advection term **v**_1_ as

(2.3) $$v_{1}(\theta )=\nu \nabla_{\theta }U(\theta ),$$

where *U*(*θ*) can be computed from the homeostasis distribution *u_s_*(*θ*) that can be regarded as the cell landscape that the hematopoiesis system desires to maintain. As in the Boltzmann-like distribution from equilibrium statistical mechanics [17], we compute *U*(*θ*) as the exponent of *u_s_*(*θ*) in the exponential form, in other words, $U\left(\theta \right)=-\ln\left(u_{s}\left(\theta \right)\right)$. There are multiple methods to compute the cell landscape, so called quasi-potential, that focuses on relative stability of multiple attractors and models cell differentiation as transition between the attractor states [6–11]. Here, we compute the cell landscape empirically by assuming that the entire single-cell data is a representative subset of the entire hematopoiesis system, and by using density approximation methods. In particular, we use kernel density estimation [18] from the projected single-cell data *θ^i^* ∈ Γ, i.e., $u_{s}\left(\theta \right)=\frac{1}{N}\sum_{i=1}^{N}K_{h}\left(\theta -\theta^{i}\right)$ where we chose the standard normal density function as the kernel function *K_h_* with bandwidth parameter *h* > 0.

The second candidate **v**_2_ models the dynamics of cell state transition. We model this term using a mechanistic approach that describes the symmetric and asymmetric cell division of stem cells to more differentiated cells. In particular, we consider the following form

(2.4) $$v_{2}(t,\theta )=c(\theta )[2(1-a(\theta ))r(\theta )]s(t),$$

that is parameterized by the proliferation rate *r*(*θ*) and the self renewal rate *a*(*θ*) [19]. $c\left(\theta \right)\in R^{n}$ represents the direction and magnitude of differentiation on the phenotype space that we can estimate with either temporal data or pseudotime inference methods [4]. We note that the self renewal rate *a*(*θ*) is the proportion of cells that remains in cell state *θ*, while 1–*a*(*θ*) cells further differentiate into matured states. This can be counted from symmetric and asymmetric stem cell division. In addition, we assume a signal parameter *s*(*t*) that controls the active differentiation term, where *s*(*t*) = 1/(1 + *km*(*t*)) and *m*(*t*) is the number of matured cells. Finally, we comment that the directed cell transition is simulated as *V* = **v**_1_ + **v**_2_, that is a sum of cell transition to attain homeostasis and active cell differentiation.

The reaction term represents the growth rate that consists of proliferation and apoptosis. We comment that the calibration of this term requires additional data to scRNA-seq, particularly, the population level growth data, to uniquely calibrate the model. It has been studied that the cell dynamics cannot be uniquely determined without imposing the reaction term [20]. More recently, there has been efforts to estimate the proliferation rate directly from scRNA-seq data by cellular barcoding techniques [21]. In our simulations, we cluster the single-cell data into biologically well known cell types, for instance, in case of hematopoiesis, myeloid progenitors, lymphoid progenitors, macrophages, and obtain the proliferation rate and self renewal rate from the literature. We consider the logistic growth term as following

(2.5) R(θ,u)=r(θ)(1−d(θ,u))u,

where *r*(*θ*) is the proliferation rate and *d*(*θ*) is the apoptosis term assuming a logistic growth as $d\left(\theta ,u\right)=\min\left\{\frac{u}{u_{s}\left(\theta \right)},\bar{d}\right\}$, where $\bar{d}$ models the maximum magnitude of apoptosis rate.

The second-order diffusion term represents the instability on the phenotypic landscape of the cells that should be taken into account when modeling the macroscopic cell density. We simply consider a constant term *D*(*θ*) = *v*. Assuming that the cell state trajectory is subject to Gaussian white noise, the diffusion coefficient can be estimated as the variance of the cell trajectory *θ*(*t*) on the reduced space, *ν* = Var(*θ*(*t*))/4. However, since we do not have the data of cell trajectories, one can estimate the value as a limit of random walk as *ν* = (Δ*x*^2^)/(4Δ*t*), assuming that Δ*x* is the step size of the phenotypic fluctuation in Δ*t* time [22]. See Appendix A for the detail of the model.

### 2.3. PDE model of cell state-transitions solved on a graph

Although the continuum-based multi-dimensional model provides a framework to study cell states, it is not always straightforward to map back locations in the space to novel or otherwise unknown cell states. Moreover, a central feature of contemporary analysis of scRNA-seq data is clustering and inferring relationships between clusters of known cell types [4]. Therefore, we develop a model that can describe cell state-transition dynamics on a graph that represents relationships between known cell types identified with clusters, extended from our previous work in [12]. An immediate advantage of this cell state geometry is that it is convenient to employ biological insights from well-known classical discrete cell states.

The continuum of differentiation cell states is assumed to be on the graph obtained from the scRNA-seq data, for instance, using partition-based graph abstraction (PAGA) algorithm [23]. We project the graph on the reduced component space, and denote the nodes as ${\left\{v_{k}\right\}}_{k=1}^{n_{v}}$ and the edges as *e*_*ij*_ connecting in the direction from the *i*-th to the *j*-th node. For convenience of notation, the edges are also denoted as ${\left\{e_{k}\right\}}_{k=1}^{n_{e}}$ with the index mapping $I:J\to \left\{1,\ldots ,n_{e}\right\}$ on the set of nontrivial edges (i,j)∈J. The end points in the direction of cell transition are ${\left\{a_{k}\right\}}_{k=1}^{n_{e}}$ and ${\left\{b_{k}\right\}}_{k=1}^{n_{e}}$, where $\cup_{k=1}^{n_{e}}\left\{a_{k},b_{k}\right\}={\left\{v_{k}\right\}}_{k=1}^{n_{v}}$.

The model follows the dynamics of the cell distribution on the graph, *u*(*x, t*), where *x* ∈ *e_k_* is a one-dimensional variable that parameterizes the differentiation continuum space location along the edges. We annotate the cell distribution on each edge *e_k_* as *u*_*k*_(*x, t*) such that $u(x,t)={\left\{u_{k}(x,t)\right\}}_{k=1}^{n_{e}}$, and model the cell density by an advection-reaction-diffusion equation [24] as

(2.6) $$\frac{\partial u_{k}}{\partial t}=-\frac{\partial }{\partial x}\left(V_{k}(x)u_{k}\right)+R_{k}(x)u_{k}+\frac{\partial }{\partial x}\left(D_{k}(x)\frac{\partial u_{k}}{\partial x}\right),\;x\in e_{k}=\bar{a_{k}b_{k}}.$$

The three terms are similarly modeled as the multi-dimensional model in Eq (2.2), representing cell differentiation, population growth, and phenotypic instability. To summarize once more, the advection coefficient *V_k_*(*x*) models the cell differentiation and the transition between the nodes, that is, different cell types. We model the advection term in two parts as in the reduced component space model, *V_k_*(*x*) = *v*_*k*,1_(*x*) + *v*_*k*,2_(*x*), and compute them as

(2.7) $${\text{v}}_{k,1}(x)=v\partial_{x}U_{k}(x),\;{\text{v}}_{k,2}(t,x)=\left[2\left(1-a_{k}(x)\right)r_{k}(x)\right]s(t).$$

Here, *u_s,k_*(*x*) = *e*^−*U_k_*(*x*)^ is the homeostasis cell distribution on the *k*-th edge, *ν* is the magnitude of phenotypic instability, *r*_*k*_(*x*) is the proliferation rate, *a_k_*(*x*) is the self-renewal rate, and *s*(*t*) is the signal parameter. Cell proliferation and apoptosis can be modeled by the reaction coefficient *R_k_*(*x*) as

(2.8) $$R_{k}(x,u)=r_{k}(x)\left(1-d_{k}(x,u)\right)u.$$

Finally, the diffusion term that represents phenotypic fluctuation is taken as *D*_*k*_(*x*) = *ν*.

In addition to the governing equation on the edges, the boundary condition at the nodes are critical when describing the dynamics on the graph. The boundary condition on the cell fate PDE model can be classified into three types, the initial nodes that do not have inflow ${\text{N}}_{I}≐\left\{v_{k}\notin \cup_{j=1}^{n_{e}}\left\{b_{j}\right\},k=1,\ldots ,n_{v}\right\}$, e.g., stem cells, the final nodes without outflow ${\text{N}}_{F}≐\left\{v_{k}\notin \cup_{j=1}^{n_{e}}\left\{a_{j}\right\},k=1,\ldots ,n_{v}\right\}$, e.g., the most differentiated cells, and the intermediate nodes, ${\text{N}}_{T}≐\left\{\cup_{j=1}^{n_{e}}\left\{a_{j}\right\}\right\}\cap \left\{\cup_{j=1}^{n_{e}}\left\{b_{j}\right\}\right\}$. On the intermediate nodes *v*_*n*_ ∈ N_*T*_, mixed boundary condition is imposed for continuity of the density and flow as following,

(2.9) $$\underset{\left(i,n\right)\in J}{\sum }{ℬ}_{I\left[i,n\right]}\left(u,b_{I\left[i,n\right]}\right)=\underset{\left(n,j\right)\in J}{\sum }{ℬ}_{I\left[n,j\right]}\left(u,a_{I\left[n,j\right]}\right),\;u\left(b_{I\left[i,n\right]}\right)=u\left(a_{I\left[n,j\right]}\right),\;\text{for all}\;\left(i,n\right)\in J,\left(n,j\right)\in J,$$

where ${{ℬ}_{I[i,j]}(u,x)≐V_{I[i,j]}(x)u(x)-D_{I[i,j]}(x)\frac{\partial }{\partial x}u(x)|}_{x_{I\left[i,j\right]}}$, *b*_*I*[*i,n*]_ is the right end point of the edge between nodes *i* and *n*, and *a*_*I*[*n, j*]_ is the left end point of the edge between nodes *n* and *j*. The cell outflow boundary conditions on the final nodes, *v_n_* ∈ N_*F*_, are imposed as reflecting boundary conditions

$$\underset{(i,n)\in J}{\sum }{ℬ}_{I[i,n]}\left(u,b_{I[i,n]}\right)=0,$$

and *u*(*b*_*I*[*i,n*]_) = *u*(*b*_*I*[*j,n*]_) for all (*i, n*) and (*j, n*) in J, and similarly on the initial nodes *v_n_* ∈ N_*I*_.

### 2.4. Quantification of cell state-transition dynamics

Let us define some useful quantities to interpret model predictions in the multi-dimensional cell state-space and on a graph. The total number of cells from the cell distribution on either a graph or a continuous manifold can be computed as

(2.10) $$\rho (t)≐\underset{k}{\sum }\int_{e_{k}}u_{k}(t,x)dx,\;\rho (t)≐\int_{\text{Γ}}u(t,\theta )d\theta ,$$

respectively. In addition, we compute the number of cells of a specific cell type by assigning a weight, *w*_*k*_, that corresponds to cells in the *k*-th cluster as

(2.11) $$\rho_{k}(t)≐\int_{\text{Γ}}u(t,\theta )w_{k}(\theta )d\theta ,$$

with ∑_*k*_*w_k_*(*θ*) = 1. We assign weights based on the relative cell density of each clusters estimated with kernel density estimation. In the graph model, we assign the cell states along the edge to be the cell type of the closest node, so that we can compute the number of the *k*-th node cell type as $\rho_{k}(t)≐\sum_{m=I\left(k,j\right)}\int_{a_{m}}^{\frac{a_{m}+b_{m}}{2}}u_{m}(t,x)dx+\sum_{m=I\left(i,k\right)}\int_{\frac{a_{m}+b_{m}}{2}}^{b_{m}}u_{m}(t,x)dx$.

Although we can understand the continuum of cell states by mapping cells in intermediate states back to known discrete cell types, we also desire to interpret the continuous cell states in their location without reference to the canonical cell identities. For such purpose, we characterize cell states by identifying genes that are strongly correlated to a location in the space or moving in a direction toward a cell state. This extends finding the genes that are correlated to specific reduced space components to analyze the reduced cell state space [25]. First, to characterize the cell state θ* in the reduced space, we use a function *f*_θ*_(*θ*) centered at θ* as $f_{\theta^{*}}(\theta )=\frac{1}{\sqrt{2\pi }\sigma }\exp\left[-{\left\|\theta -\theta^{*}\right\|}^{2}/2\sigma^{2}\right]$, and compute the correlation between the function values and the *j*-th gene expression levels as

(2.12) $$r_{f,j}≐\text{corr}\left(f,g_{j}\right),$$

where ***f*** represents the vector of function evaluation at each single-cell data point *θ*^*i*^, that is, $f={\left\{f_{\theta^{*}}\left(\theta^{i}\right)\right\}}_{i=1}^{N}$ and $g_{j}={\left\{g_{j}^{i}\right\}}_{i=1}^{N}$. An alternate quantity to examine is the genes that are related to a certain direction in the reduced component space. For instance, the correlation between the *j*-th gene and the *k*-th reduced component $\theta_{k}={\left\{\theta_{k}^{i}\right\}}_{i=1}^{N}$ and to a certain vector $v={\left\{v_{k}\right\}}_{i=1}^{n}$ can be computed as

(2.13) $$r_{k,j}≐\text{corr}\left(\theta_{k},g_{j}\right),\;r_{v,j}≐\underset{k}{\sum }v_{k}r_{k,j},$$

respectively. Regarding Eq (2.13) as global quantities, we can also compute the local correlation on the subdomain of the reduced space Ω_*d*_ by collecting the cell indices that lie within the subdomain ${\text{Γ}}_{d}=\left\{i|\theta^{i}\in {\text{Ω}}_{d}\right\}$, that is, $r_{k,j}{|}_{{\text{Γ}}_{d}}≐\text{corr}\left(\left(\theta_{k},g_{j}\right)|{}_{i\in {\text{Γ}}_{d}}\right)$ and $r_{v,j}{|}_{{\text{Γ}}_{d}}≐\sum_{k}v_{k}\rho_{k,j}|{}_{{\text{Γ}}_{d}}$. Although these metrics may provide candidates of potential genes that are related to the cell state to be analyzed, we emphasize that these need to be verified experimentally by observing the cell state change after perturbing the genes. See Section 4.2 for the limitations of this approach.

## 3. Simulation of continuous cell state models on multi-dimensional space versus graph

In this section, we employ the framework developed in the previous section to the mouse hematopoiesis cell data from Nestorowa et al. (2016) [13] and Paul et al. (2015) [14]. We obtain the graph and multi-dimensional space models of hematopoiesis cell state and focus on comparing the strengths and weaknesses of the two models.

**Figure 2.**
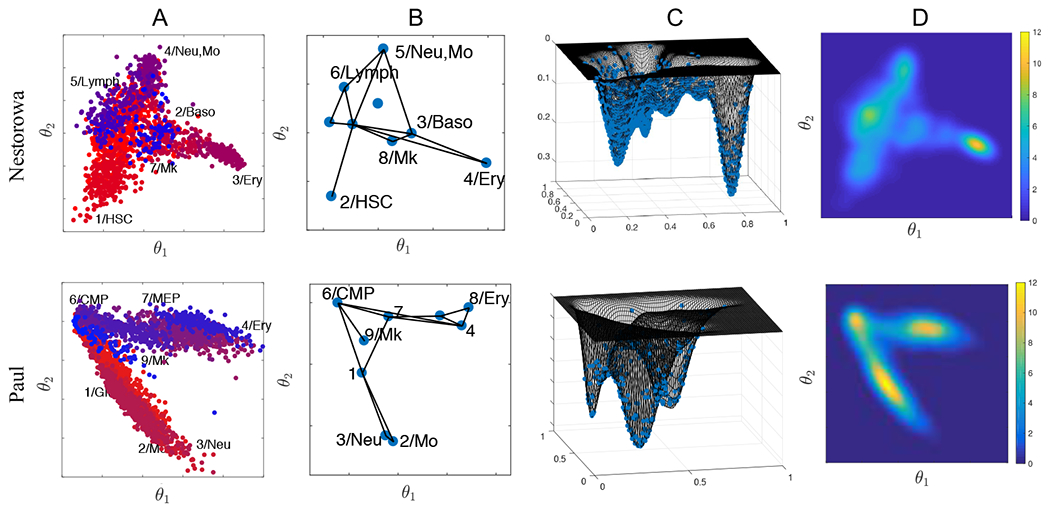
From discrete to continuum cell states. A) Single-cell data from Nestorowa et al. (2016) and Paul et al. (2015) projected on the first two diffusion component space. B) Graph obtained by PAGA algorithm projected on the diffusion component space. Distinct cell types classified in the original paper are either illustrated with different colors (A) or annotated on the graph nodes (B). C, D) Multi-dimensional continuum cell state distribution on the diffusion component space computed by kernel density estimation. They are used as homeostasis distribution.

The hematopoiesis single-cell data from [13, 14] projected on the first two diffusion component space are shown in Figure 2A, where distinct cell types, including lymphoid-primed multipotent progenitors (Lymph); common myeloid progenitors (CMP); megakaryocyte-erythroid progenitors (MEP); granulocyte-macrophage progenitors (GMP); erythrocytes (Ery); neutrophils (Neu); monocytes (Mo); megakaryocytes (Mk); basophils (Baso), classified in the original papers are illustrated with different colors. We truncate the diffusion component at two since the reduced two-dimensional space describes the dynamics of our interest, that is, from strong to weak stemness. The first two diffusion components *θ*_1_ and *θ*_2_ represent cell maturation in both data sets. In Nestorowa data, the first diffusion component separates the stem cells to myeloid lineages, particularly MEP cells and the second diffusion component describes GMP cells and the lymphoid lineages. In Paul data, the first and second reduced component represents MEP and GMP lineage, respectively. We remark that the cells that are the most stem-like in Paul data are CMPs, that is more matured than the ones in Nestorowa data, that includes the long-term and short-term HSCs. In addition to the single-cell data, the Figure 2B presents the abstracted graphs obtained using PAGA [23]. Further refinements of the graph will eventually become the full single-cell data, where each single-cell being counted as distinct cell states, and it depicts the hierarchy of cell states (see Figure A5).

The homeostatic cell distribution *u*_*s*_ on the reduced dimensional space is computed by kernel density approximation [18, 26]. The computed cell landscapes viewed in different angles are shown in Figure 2C,D. The cell distribution on the graph can be similarly obtained after reallocating the cells to the node, that is, the center of each cluster. The cell distribution on the continuum space provides an intuitive method to compare the relative concentration of different cell lineages, including the intermediate cell states. We observe high concentration of MEP and Ery cells that are localized at the far right (large *θ*_1_) in both data. The Nestorowa data has more diverse cells including the common lymphoid progenitors that are visible on the left (small *θ*_1_, and intermediate *θ*_2_), while the Paul data has evenly distributed cell states among the most stem-like cells (CMP) and the two different lineages.

Let us summarize the properties of the graph and multi-dimensional space models before we present simulation results. The graph model has its strength that the cell lineages between the known cell states can be more easily identified as compared to the multi-dimensional space model. The cell concentration moving toward different edges can be clearly distinguished as the cell lineages to different cell states. Accordingly, counting the number of cells in each discrete cell state is more straightforward, for instance, by integrating the cell distribution along the edges half way. Although the multi-dimensional space model has ambiguity on classifying the cells into discrete cell types, the number of cells in each discrete cell type can be computed by assigning weights to integrate as in Eq (2.11). Moreover, we emphasize that the advantage of clear cell states in the graph model is also its limitation at the same time, since it restricts the model to only study the known cell types and lineages. The advantage of the multi-dimensional space model is its potential of exploring novel cell states that deviates from known cell types. While the graph model cannot explore the cell states that are not already included in the graph structure, the multi-dimensional space model can immediately study the abnormal trajectories and emergence of cells at any space location. We will show later in our simulation that the hypothesis of genetic alterations can be studied directly in the multi-dimensional space model, without projecting it on the graph structure. Moreover, the multi-dimensional space model is more sensitive to genetic variations than the graph model, although when the variation is large and a considerably distinct cell state arises, the graph model can append another cluster node. See Table 1 for a summary.

In the following sections, we consider mainly two application problems, namely, normal hematopoiesis and abnormal hematopoiesis differentiation, resulting in myeloid leukemia as application examples of our modeling approach.

### 3.1. Calibrating the mathematical models to normal hematopoiesis

**Figure 3.**
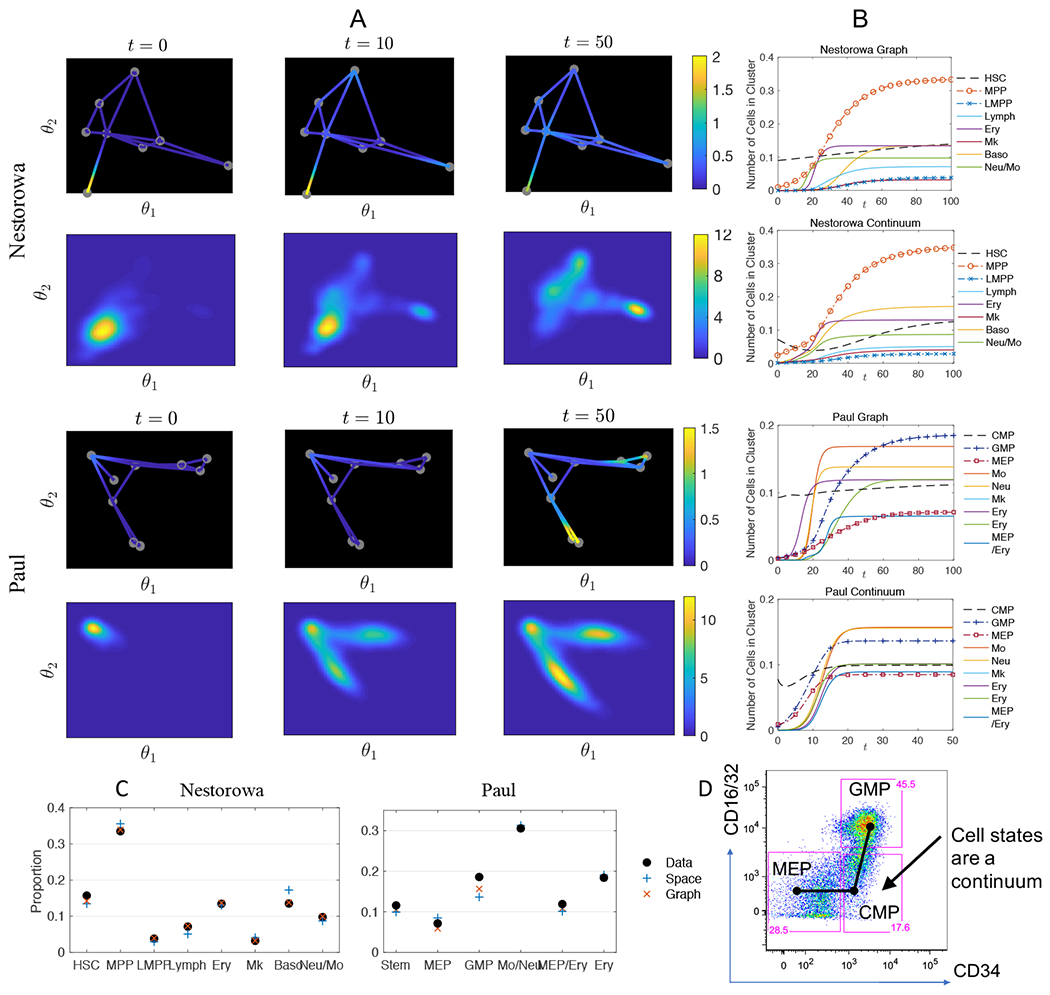
Dynamics of cell distribution during normal hematopoeisis. A) Evolution of cell state densities *u*(*t, x*) on the graph with 8 to 9 nodes, and *u*(*t, θ*) on the diffusion component space during normal hematopoeisis using single-cell data from Nestorowa et al. (2016) and Paul et al. (2015). The shown dynamics is in pseudotime *t*. B) The pseudotime dynamics of the number of cells in each cell cluster, where the number is normalized so that the total cell number in equilibrium state is one. The initial stem cells differentiate to progenitors and more matured cell states and recover the entire cell landscape. C) Numbers of cells in each type/cluster using the multi-dimensional space model and the graph model are successfully calibrated to the observed data so that at *t* = 100 each model predicts the correct cell ratios to within ±5%. D) The continuous cell states of hematopoeisis is also depicted in the FACS data set collected from the normal mouse bone marrow. Bone marrow cells were gated for myeloid progenitor cell markers (lineage-negative, Sca1-negative, cKit-positive). Conventionally, the expression levels of CD16/32 and CD34 are used to distinguish CMP, GMP, and MEP cell types within the myeloid progenitor compartment, however, the continuity of marker expression agrees with our graph abstraction and multi-dimensional cell state geometries.

**Figure A1.**
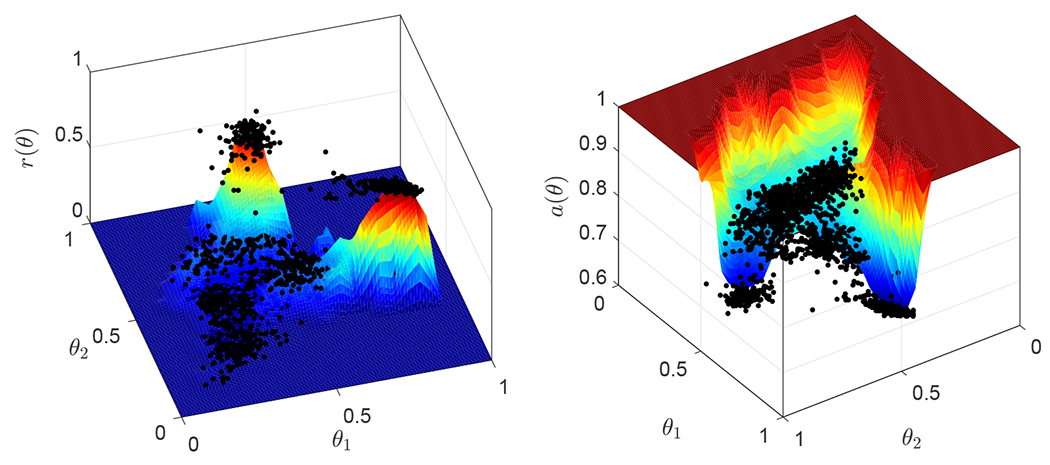
Cell proliferation rate *r*(*θ*) and self-renewal rate *a*(*θ*) computed from the single-cell data. The black dots are the rates of data.

**Figure A2.**
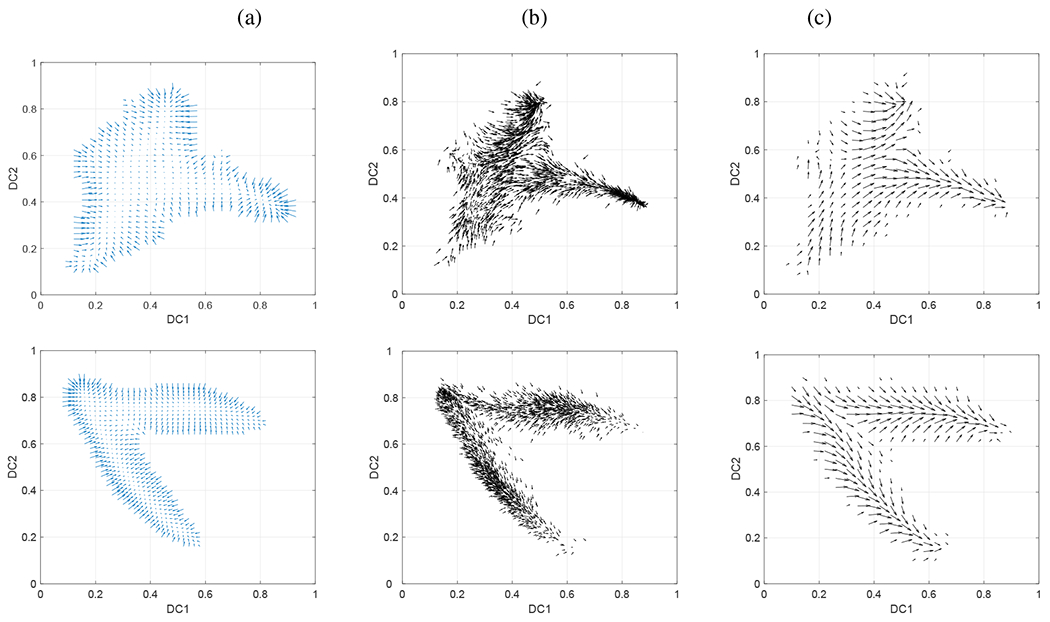
Pseudotime dynamics. The homeostasis cell differentiation vector **v**_1_ (a), and the direction of active cell differentiation obtained from diffusion pseudotime analysis (b), and that interpolated at the grid points **v**_2_ (c) are presented. We remark that, **v**_2_ corresponds to the cell differentiation along the edges in the graph model.

**Figure A5.**
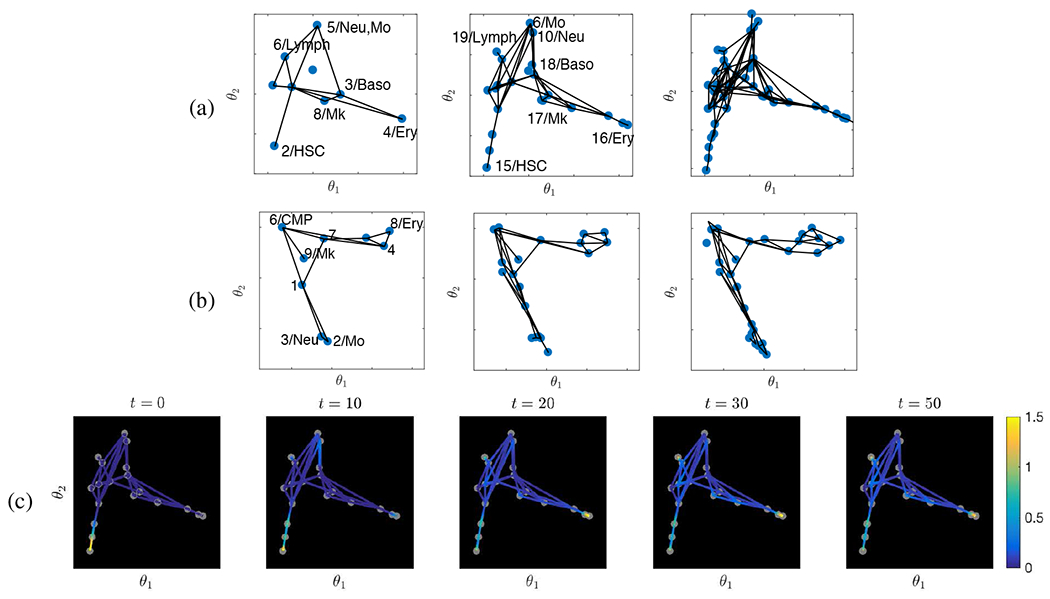
From discrete to continuum cell states. The hierarchy of graphs using partition-based graph abstraction [23] and single cell data from Nestorowa et al. (2016) (a) and Paul et al. (2015) (b). The single-cell data can be regarded as the most refined graph. The simulation of normal hematopoiesis on graph with 19 nodes (c), that is comparable to Figure 3A, illustrates the hierarchy of cell distribution toward the entire reduce space.

We demonstrate that normal hematopoiesis can be visualized by both models on the graph and on the space of two-dimensional diffusion components, (see Figures A1 and A2 for the advection and reaction terms used in the multi-dimensional space model). Figure 3 shows a cluster of stem cells differentiating into the entire cell states on the graph and reduced space using Nestorowa data [13] and Paul data [14]. The initial condition is imposed as approximately 10% of cell capacity in normal condition mostly composed with stem cells. On both graph and multi-dimensional space models, nontrivial amount of most matured cell states, particularly, Ery and Neu/Mo cells arise around pesudotime *t* = 30, and further recovers the full landscape after approximately *t* = 50. In particular, the observation that the matured cells quickly proliferate to fill in the space agrees in both simulations from Nestorowa and Paul, while the effect is more significant in Paul’s data due to shorter distances of the matured cell states from the initially administered cells.

**Figure A6.**
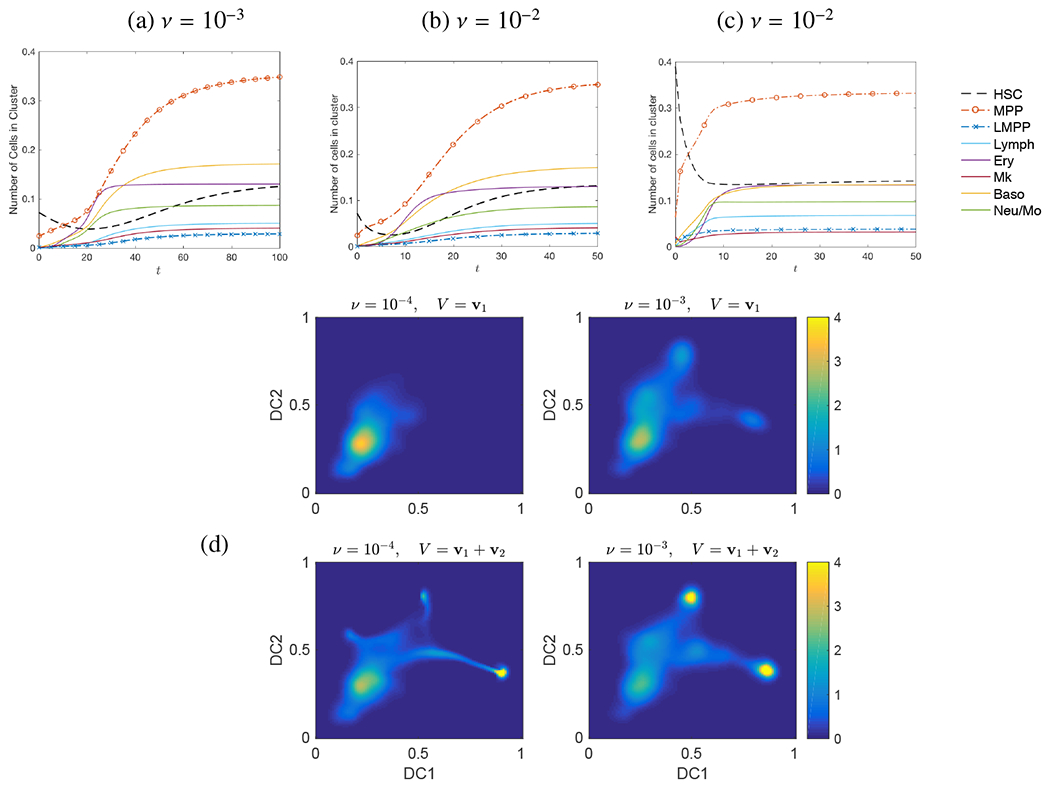
Model sensitivity to parameters. Using single cell data from Nestorowa et al. (2016), number of cells and its dynamics in each cluster up to *t* = 50 for different values of *ν* and initial stem cell numbers *ρ*(0) are shown in (a–c). The dynamics of cells in each cluster for *ν* = 10^−3^ with *ρ*(0) = 0.1 (a), *ν* = 10^−2^ with *ρ*(0) = 0.1 (b), and *ν* = 10^−2^ with larger initial number of cells, *ρ*(0) = 0.5 (c) shows that the recovery is more rapid for larger values of *ν* and larger number of initial stem cells *ρ*(0). Cell distribution *u*(*θ*, *t*) at intermediate time *t* = 14 for advection terms **v**_1_ and **v**_1_ + **v**_2_, and *ν* = 10^−4^ or *ν* = 10^−3^ are shown in (d). The distributions are distinct, where larger values of *ν* increases overall rate of differentiation, while adding **v**_2_ prioritizes recovery of the most matured cells.

The advantage of the graph model is apparent that we can observe distinct cell states as a mass of cells shifting from a node to distinct edges toward different cell states. For instance, the cells differentiating from the MPP cluster to either Neu/Mo lineage and Ery lineage can be clearly separated in the graph models, while those can be ambiguous in the two-dimensional distribution. Still, we can compute the number of cells in each cell types in both models as shown in Figure 3B. We observe that the number of cells reaches the maximal capacity at later times around *t* = 100, with the intended ratio of cell numbers in each discrete cell type approximating the given data [13] in Figure 3C. The recovery is more rapid for larger values of *ν* and larger number of initial stem cells *ρ*(0) (see Figure A6). We remark that the continuous cell states of hematopoeisis is also depicted in conventional flow cytometry which is typically used to identify distinct cell populations based on expression of cell surface markers. We performed Fluorescence Activated Cell Sorting (FACS) analysis of bone marrow cells isolated from normal C57Bl/6 mice (age 6-8 weeks). Distinct myeloid progenitor types (CMP, GMP and MEP) are typically differentiated by the expression of CD16/32 and CD34 markers within the myeloid lineage progenitor cell compartment. Figure 3D shows representative FACS data with respect to CD16/32 and CD34 expression that is used to identify the CMP, GMP, and MEP cell types within the normal myeloid progenitor compartment. Although the FACS data is conventionally clustered and gated into three cell types, continuity of CD16/32 and CD34 expression can be observed that agrees with our graph abstraction and multi-dimensional cell state geometries.

### 3.2. Using the model framework to simulate acute myeloid leukemia (AML) pathogenesis and progression

In this section, we once more compare the graph and multi-dimensional space models with an application to abnormal differentiation under leukemia pathogenesis and progression. We first consider AML model in the context similar to the previous section that involves known progenitor cells that exemplifies the advantage of graph models. However, we will show how aberrant differentiation and phenotypic plasticity of leukemia pathogenesis motivates the spatial model.

**Figure 4.**
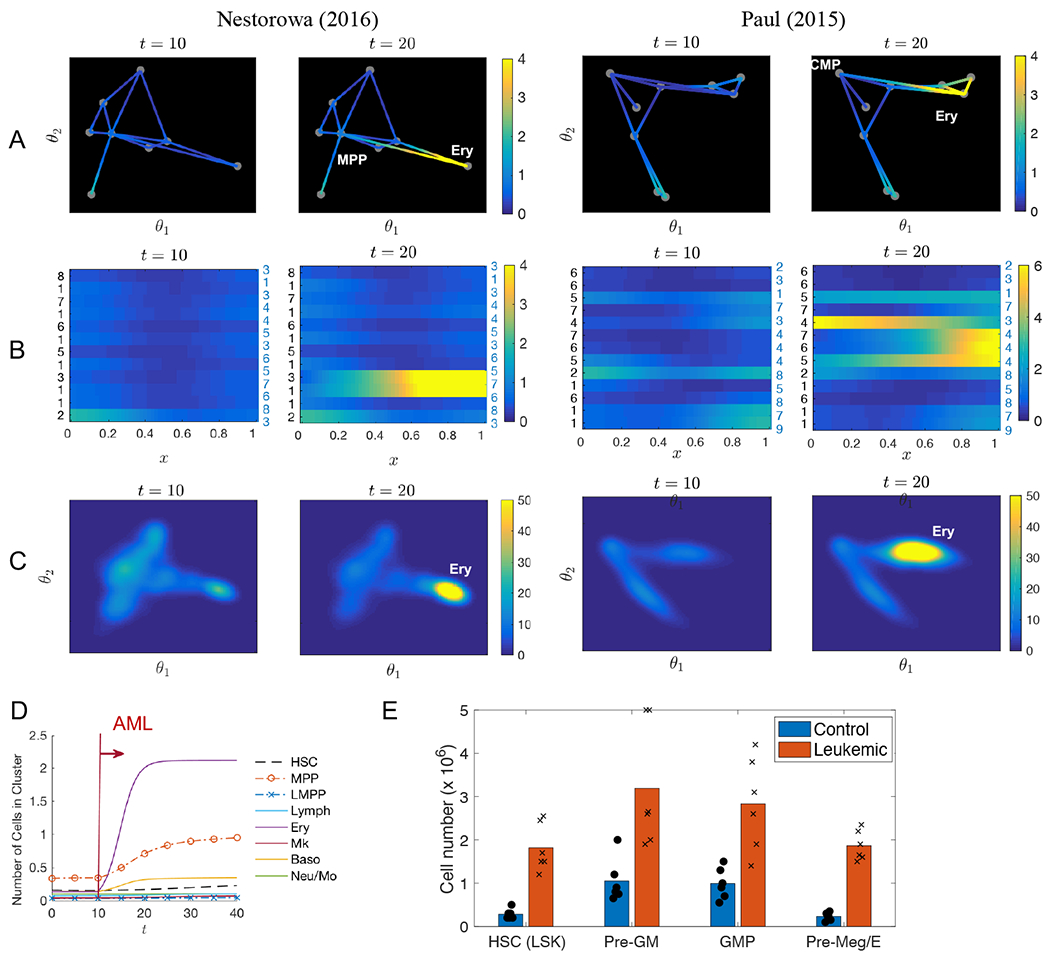
Predicting abnormal cell differentiation during leukemia progression. A,B,C) Cell distributions during leukemia pathogenesis and progression are shown on the graph model (A, B) and the multi-dimensional space model (C). Plot (B) shows an alternative way to plot the graph based model solution by stacking the cell distribution on each edge horizontally. The number on the left and right shows the node numbers shown in Figure 2B. They show the effect of over-proliferation and differentiation block in the myeloid lineages. In particular, we observe a rapid expansion of cell states near MEP and Ery in both Nestorowa and Paul data, after the initiation of AML at *t* = 10. D) The number of leukemic Ery cells show an increase within ten days in both graph and multi-dimensional space models. E) Experimental result reproduced from [27] that shows a rapid expansion of pre-Meg/E (MEP) population in leukemic mouse compared to normal mouse (Control).

AML results from aberrant differentiation and proliferation of transformed leukemia-initiating cells and abnormal progenitor cells. We model AML pathogenesis based on known behavior of a genetic Cbfb-MYH11 (CM) knock-in mouse model that recapitulates somatic acquisition of a chromosomal rearrangement, inv(16)(p13q22) [28, 29], commonly found in approximately 12 percent of AML cases. Inv(16) rearrangement results in expression of a leukemogenic fusion protein CBF*β*-SMMHC, which impairs differentiation of multiple hematopoietic lineages at various stages [30–32]. Most notable in such leukemia pathogenesis and progression is the increased in abnormal myeloid progenitors, which has an MEP-like immunophenotype and a CMP-like differentiation potential [31]. Experimental studies [27, 33] show that such MEP attains a predominant increase in pre-megakaryocyte/erythroid (Pre-Meg/Ery) population (ranging from 5 to 12 fold) accompanied by impaired erythroid lineage differentiation as shown in Figure 4E. This refined phenotypic Pre-Meg/Ery population consists partly of the CMP and MEP populations which are identified using conventional markers [13, 34].

In our model, abnormal leukemic progenitors are regarded as intermediate cell states along the edges connecting CMP (or MPP) and MEP (and Ery) in the graph model, and the corresponding locations in the multi-dimensional space model. We assume a 10-fold increase on average in those population by lowering *d*(*θ*) and *d*_*k*_(*x*) in Eqs (2.5) and (2.8) that controls the local cell capacity. In addition to over-proliferation, another aspect of the leukemia pathogenesis of our interest is the impaired differentiation of erythroid lineage differentiation, where it can be modeled by blocking the cell differentiation *V*(*θ*) in Eq (2.6) and *V*_*k*_(*x*) in Eq (2.2) toward Ery.

The corresponding results are shown in Figure 4, where we modify the model to leukemia progression at *t* = 10. The cell distribution changes from the normal cell landscape at *t* = 10 to an increased population of Ery (MEP) and nearby cells at *t* = 20 in both graph and continuum models. We observe a 10-fold increase in the Ery (MEP) population, which includes the abnormal myeloid progenitors, in both graph and multi-dimensional space model across the data sets as shown in Figure 4D,E. The rapid emergence of AML occurs within two week period, corresponding to the expansion of the leukemic cell phenotype. We observe a rise in the MPP or CMP cluster as shown in the results from Nestorowa data, that is similar in Paul data as well. The total proportion of leukemic cells comprise 50–60% of the total population.

In the leukemia pathogenesis simulation in this section, we focus on studying the leukemic cells as a variation of cell states classified using conventional markers. In this case, the graph model can interpret and include the dynamics of such cells, as well as the multi-dimensional space model. While the simulation outcome between the graph and multi-dimensional space model is similar, the graph model is computationally more efficient due to the fewer number of unknowns as compared to the two-dimensional space model. However, to study the abnormal cell states that may appear far away from the known or existing landscape, we will show in the following section that the multi-dimensional space model has more freedom to include those new cell states and disrupted trajectories. We will study the impact of perturbation of genes in the graph and multi-dimensional space model, particularly focusing on alterations of genes known to be involved in leukemia pathogenesis.

### 3.3. In silico experiments of gene expression perturbation

**Figure 5.**
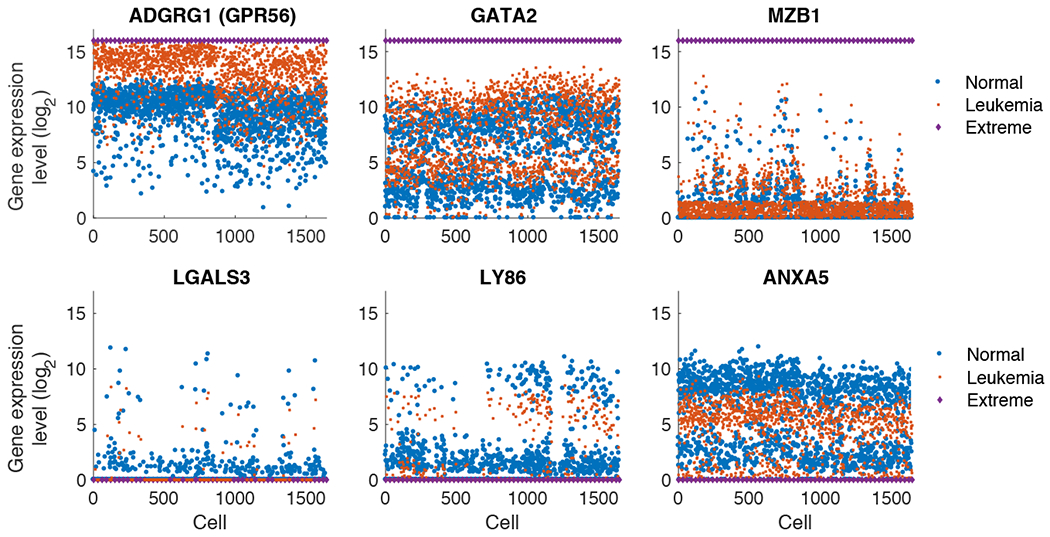
Perturbing genes associated with leukemia stem cells. Examples of expression levels of genes in log_2_ scale, that are associated with leukemia stem cells and pathogenesis, including up-regulated GPR56, GATA2, and MZB1, and down-regulated LGALS3, LY86, and ANXA5. We show these subset of genes simply to illustrate the process. The normal single-cell data $\log_{2}\left(g_{j}^{i}+1\right)$ (blue circle) and modified gene expression $\log_{2}\left({\tilde{g}}_{j}^{i}+1\right)$ (red square) computed as Eq (3.1) are shown together, with the case of extreme levels of either 16 or 0 (purple diamond).

In this section we investigate the sensitivity of altering specific genes in a prescribed manner and the impact of this perturbation in the graph and multi-dimensional space models. We keep our focus on leukemia pathogenesis and progression and consider alterations of 38 genes that are reported to be related to leukemia stem cells [35, 36], although we emphasize that these genes serve simply as examples, and are not intended to model the precise biological process of AML pathogenesis. The *j*-th gene expression level of *i* cell, $g_{j}^{i}$, is modified as

(3.1) $${\tilde{g}}_{j}^{i}=2^{\gamma_{j}}g_{j}^{i},\;0\leq \log_{2}\left({\tilde{g}}_{j}^{i}+1\right)\leq 16,$$

where *γ*_*j*_ is the log_2_-fold change compared to normal cells. The full list of altered genes and magnitudes *γ*_*j*_ from [35, 36] are shown in A1. Details of the model equation and parameters, and the log_2_-fold change is in the range of *γ*_*j*_ ∈ [−3.5, 2.7]. In addition, we consider the extreme case of gene alteration as the maximum level $\log_{2}\left({\tilde{g}}_{j}^{i}+1\right)=16$ for up-regulated genes and $\log_{2}\left({\tilde{g}}_{j}^{i}+1\right)=0$ for down-regulated genes. Figure 5 shows examples of the gene expression levels in log scale that we modify including the up-regulated genes, GPR56, GATA2, and MZB1, and the down-regulated genes, LGALS3, LY86, and ANXA5. The given single-cell data in normal condition is plotted together with the hypothetically altered levels of gene expressions in regular leukemia pathogenesis and extreme levels of alteration. Although the case of extreme alteration is unrealistic, we consider such case to illustrate an example where the graph abstraction and dimension reduction algorithm clearly distinguishes the leukemic cells.

### 3.4. Effects of gene perturbation on graph abstraction and multi-dimensional reduced component space

**Figure 6.**
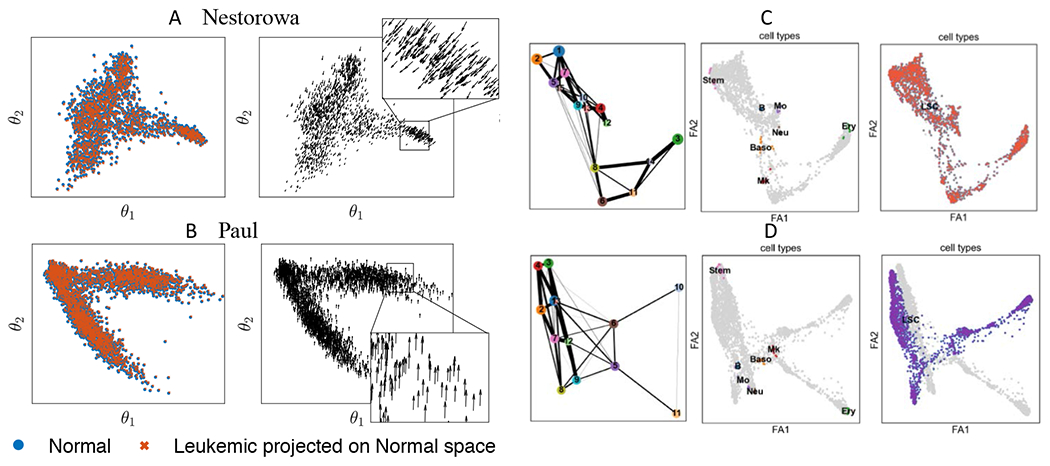
Effects of perturbing genes on cell state-space. A,B) Projection of perturbed leukemic single-cell data on the normal diffusion component space, and the directional vectors $P\left({\tilde{g}}^{i}\right)-P\left(g^{i}\right)$ representing the altered cell state by leukemic perturbation. The top figures are computed with Nestorowa data [13] (A) and the bottom figures with Paul data [14] (B). C,D) Graph computed from perturbed leukemic single-cell data and their cluster information. The annotation shows that the graph abstraction algorithm does not distinguish the perturbed leukemic cells in regular magnitude to the normal cells, so that the perturbed information is lost (C). However, when single-cell data is modified to the extreme values of gene expression level, the algorithm distinguishes the leukemic cells, although the data is unrealistic (D).

We first study the sensitivity of the reduced component space using diffusion mapping [15]. Figure 6A,B compares the altered leukemic single-cell data ${\tilde{g}}^{i}$ projected on the normal reduced space (*θ*_1_,*θ*_2_). The left-most column shows the projected leukemic single-cell data $P\left({\tilde{g}}^{i}\right)$ in the normal reduced space, where the leukemic cells are located toward the left-bottom compared to the normal cells in Nestorowa data, and upwards in Paul data. The effect of gene modification is shown more clearly in the presented vector field $P\left({\tilde{g}}^{i}\right)-P\left(g^{i}\right)$.

Similarly, we study the impact of leukemia-associated gene perturbation in graph abstraction using PAGA [23]. Figure 6C,D shows the clustered cell types and the corresponding graph using perturbed leukemic scRNA-seq data. The presented results are computed with Nestorowa data. The clustered cell types and leukemic cells are annotated to depict the cluster properties. In Figure 6C, which is the case of leukemia progression with single-cell data altered in regular magnitude, we observe that there is no cluster that separates the leukemic cells. Thus, the information of leukemic gene alteration is lost within the clustering algorithm, and the model on such abstracted graph is not capable of studying the perturbed cells. On the other hand, when the gene levels are modified to their extreme levels, the perturbed leukemic single-cell data are clustered into separate nodes as shown in Figure 6D. In this case, although the graph model is able to study the perturbed cells as separate nodes, we comment that this level of perturbation is an unrealistic scenario due to the extreme levels of gene expression.

A strength of the multi-dimensional cell state model is its capability of interpreting the perturbation of gene expression levels or new incoming cell data regardless of its relation to the primary data (Figure 6). As shown in our results, the leukemic alteration is successfully projected in the reduced space, while the abstracted graph lost the information. Although the projected directions in the reduced space can be once more projected on the graph, it does not make sense to do so when the direction is orthogonal to the edges. The multi-dimensional space model has its advantage especially in this case, where the projected direction of cell states can be directly implemented.

### 3.5. Simulating AML pathogenesis by perturbing known leukemia-associated genes

**Figure 7.**
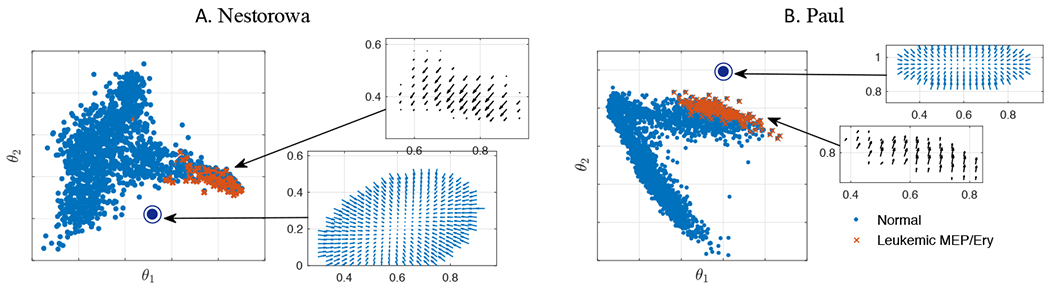
Modeling AML pathogenesis and progression by perturbing cell states directly in the cell state-space. The direction of abnormal cell differentiation $v_{aml}^{1}$ (black) is computed from the projection of altered leukemic MEP and Ery cells (×) to the normal diffusion component space as $P\left(\tilde{g}\right)-P\left(g\right)$. Alternatively, we assume a source of abnormal cell state (Θ) at θ* = (0.610, 0.215) in Nestorowa data (A) and at θ* = (0.6, 1) in Paul data (B) to model $v_{aml}^{2}$ (blue).

In this section, we incorporate the perturbed leukemia-associated gene data in the AML simulation using the multi-dimensional space model. In particular, we are interested in studying the impact of leukemia-associated gene alteration on the cell distribution during AML progression. We compute the abnormal differentiation of leukemic cells by projecting the altered single-cell data of MEP and Ery cells to the normal diffusion component space $P\left({\tilde{g}}^{i}\right)$ as it is shown in Figure 7. The aberrant differentiation vector $v_{aml}^{1}=P\left({\tilde{g}}^{i}\right)-P\left(g^{i}\right)$ shows the shifts of cells toward the location where no cell data occupies. Therefore, in addition to modifying the advection term according to the altered gene data, we assume an emergence of new abnormal cell state. In particular, we take the cell state location at θ* = (0.610, 0.215) in Nestorowa data, and at θ* = (0.6, 1.0) in Paul data, and use Gaussian functions centered at θ* to obtain $v_{aml}^{2}$. The corresponding vector fields are also shown in Figure 7.

**Figure 8.**
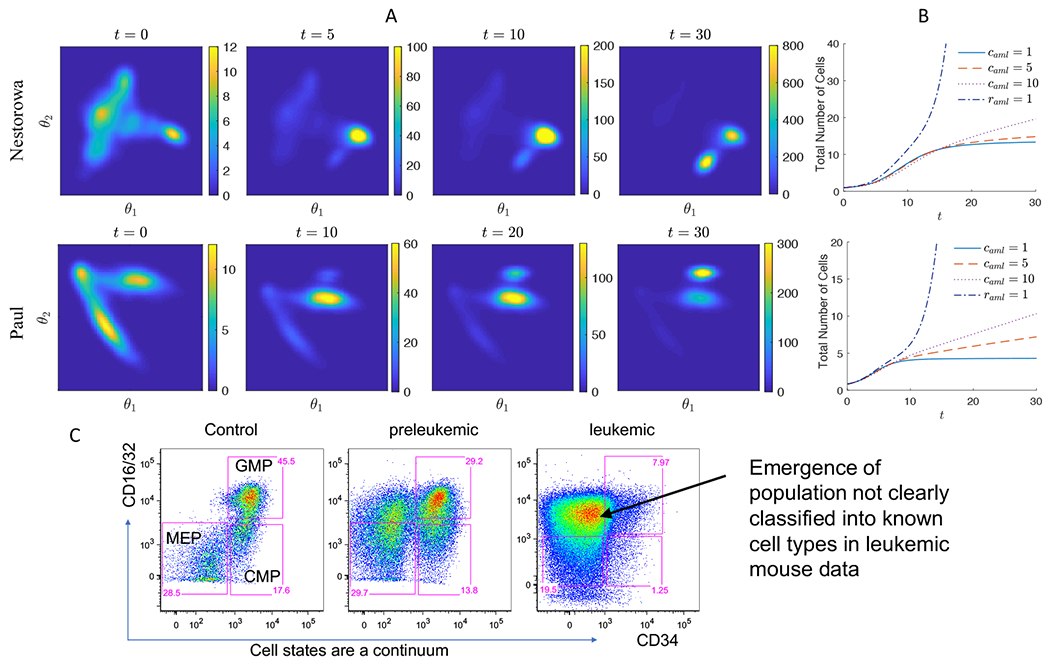
Cell state-transition dynamics during leukemia pathogenesis and progression. A) The evolution cell state distribution *u*(*t, θ*) with *c*_*aml*_ = 2. B) The total number of cells in AML condition is computed using model $V=v_{1}+c_{aml}v_{aml}^{2}$. More rapid progression of AML in terms of cell number is observed for larger values of *c*_*aml*_ and *r*_*aml*_. C) FACS analysis for CD34 and CD16/32 expression in myeloid progenitor compartment of control (left), preleukemic (center) and leukemic (right) CM knock-in mouse shows emergence of unconventional cell states during leukemic progression that eventually dominate the entire progenitor population. Our multi-dimensional cell state model is capable of incorporating those novel cell states.

For AML progression, the advection term is modeled with the prescribed vector field as $V=v_{1}+c_{aml}v_{aml}^{1}$ or $V=v_{1}+c_{aml}v_{aml}^{2}$, where *c*_*aml*_ parameterizes the perturbation magnitude. We further perturb the model by increasing the proliferation of the new leukemic cells at θ* by appending *r*_*aml*_ *f*_θ*_(*θ*) to *R*, where *r*_*aml*_ parameterizes the over-proliferation. The cell distribution *u*(*t*, *θ*) with $v_{aml}^{1}$ and $v_{aml}^{2}$ for different values of *c*_*aml*_ = 1, 2, 10 are presented in Figure 8 and Abnormal cell state transitions during leukemia pathogenesis and progression. The distribution of cell states *u*(*t*, *θ*) show abnormal cell states emerging during leukemic progression after *t* = 10 modeled in the advection term as $V=c_{aml}v_{aml}^{1}$ (top) and $V=v_{1}+c_{aml}v_{aml}^{2}$ (bottom), with various levels of *c*_*aml*_ = 1, 2, 10. Larger magnitude of *c*_*aml*_ results in more disrupted cell landscape. . In the cell landscape with $v_{aml}^{1}$, we observe increased MEP cells and abnormal progenitors arising in the direction of left-bottom, especially for large values of *c*_*aml*_. The model with $v_{aml}^{2}$, a new cell state further down in the cell space emerges and dominates the population. With the model $V=v_{1}+2v_{aml}^{2}$, new abnormal cells appear around *t* = 10 and dominate the population at *t* = 30. The total number of cells is plotted in Figure 8B, where the effects of the parameters, *c*_*aml*_ and *r*_*aml*_, are shown more clearly. The total number of cells increases more than 10 times the initial size after *t* = 30 when *c*_*aml*_ = 10 and *r*_*aml*_ = 0. When the over-proliferation term is appended as *r*_*aml*_ = 1, the total number of cells increases more rapidly, for example, up to 100 times the initial size and the number of cells in most of the myeloid lineage increases. Our simulation results agree with the experimental data, where unconventional cell states emerge during leukemia progression and eventually overtakes the entire progenitor population as observed by FACS analysis of bone marrow progenitor cells isolated from CM knock-in preleukemic and leukemic mice (Figure 8C). The predominant population observed in leukemic bone marrow does not fall within the typical gates in conventional cell clustering based on data from normal control mice (Figure 4E). Although this novel population would had been classified as MEP, pre-Meg/E, Pre-GM, and GMP cells in the graph model (Figure 4A,B), we emphasize that they are distinct population and the multi-dimensional model is capable of incorporating novel cell states. Although we comment that, in the graph model, a new cell type can be included by adding a new node to the original graph.

### 3.6. Interpretation of new cell states in the multi-dimensional model

**Figure 9.**
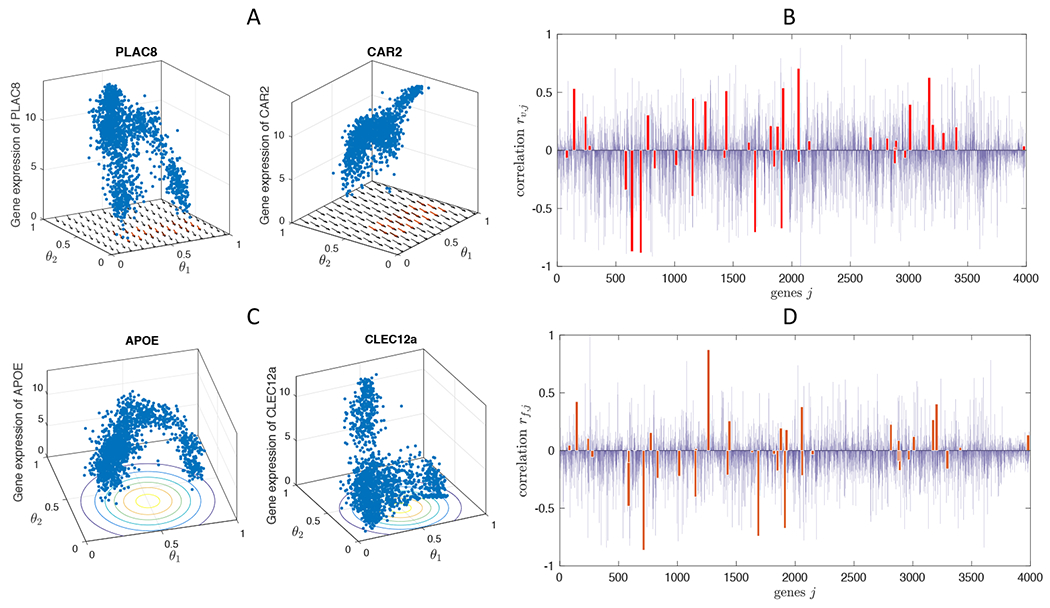
Interpretation and mapping of model-predicted novel cell states. In order to identify novel cell states predicted by the mathematical model, gene expression levels $\log_{2}\left(g_{j}^{i}+1\right)$ that are strongly correlated to the direction of leukemic alteration ***v*** = (−0.068, −0.206) (A), and to the reduced space location θ* = (0.5, 0.35) (C). The rescaled correlation *r_f,j_* (B) and *r_v,j_* (D) computed for all the genes in Nestorowa data are shown, and the leukemia related genes are marked in red bars.

The remaining question is how to interpret the new cell states in the multi-dimensional space model that may arise far away from the cell states identified by conventional markers. Hence, we propose some measures in Eqs (2.12),(2.13) to guide the interpretation. Figure 9 shows an example of the rescaled correlation quantities *r*_*f,j*_ and *r*_*v,j*_ computed with Nestorowa data. The first row show results of the correlation *r*_*v,j*_ to the average leukemic directional vector ***v*** = (−0.068, −0.206). The gene expression levels of genes that have large values of *r*_*v,j*_ are depicted in the figure, namely, PLAC8 and CAR2. We remark that those genes have strong local correlation *r_v,j_*|_Γ_*d*__ on Γ_*d*_ = {0.3 ≥ *θ*_1_ ≥ 0.9, 0.3 ≥ *θ*_2_ ≥ 0.5} as well. Figure 9 shows the correlation of all 3991 genes, where the red bars highlight the leukemia related genes we modify (A1. Details of the model equation and parameters) and we observe large magnitudes in some of the genes. The second row shows the correlation *r*_*f,j*_ to a specific cell state at the reduced space, where we choose θ* = (0.5, 0.35), which is approximately an intermediate location between MEP and CMP cells, and $f_{\theta^{*}}(\theta )=\frac{1}{2\pi 0.05}\exp\left[-{\left\|\theta -\theta^{*}\right\|}^{2}/0.1\right]$. APOE and CLEC12a genes show the largest magnitude of *r*_*f,j*_, and similarly, we can identify the leukemia related genes that show strong correlation to cell state θ*. Although more careful and rigorous approach should be developed to characterize the new arising cell states, *r*_*f,j*_ and *r*_*v,j*_ defined in Eqs (2.12),(2.13) provides an efficient method of initial screening of possible related genes.

## 4. Discussion

We have shown how to construct mathematical models of cell state-transitions using scRNA-seq data. We compare two cell state geometries: solving equations on graphs and solving equations on a multi-dimensional cell state-space. Each cell state geometry has its strengths and limitations. Selecting a model for a given application or dataset will depend on the type of biological data and the nature of the scientific question.

When the modeling application and quantity of interest includes well-known cell lineages and relation between the conventional cell states, the graph model is more appropriate due to its ability of distinguishing distinct cell lineages more clearly compared to the multi-dimensional space model. Dynamics of cell numbers in specific cell states, alteration of proliferation and apoptosis in particular cell state, differentiation block, and emergence of intermediate cell states can be quantified and studied in a straightforward manner. However, to explore cell states beyond known cell lineages, the continuum space model is more advantageous since it includes all intermediate and pathological cell states, rather than confining the model into presumed cell lineages. Moreover, the continuum model can incorporate a relatively small genetic and epigenetic alteration that the graph abstraction may not recognize, and study abnormal trajectories that yield unconventional cell states.

We selected and perturbed genes to simulate AML based on genes known to be associated with leukemia pathogenesis. We do not intend for this to be an accurate model of the biological process, rather, as an illustration of how one may select sets of genes and perturb them in a prescribed fashion in order to study the effect on cell state-transition dynamics. This approach assumes that AML pathogenesis originates from changes in gene expression in specific cell subsets, which is limited by our identification of these genes based on published literature. We acknowledge this is a limitation of the modeling approach, although we also note that our model predictions are consistent with known features of AML progression.

### 4.1. Comparison to other approaches

Although at the time of this work there are relatively few mathematical models published which utilize single-cell sequencing data, there are a few notable exceptions. Of particular note are works which use modeling and simulation to generate synthetic *in silico* gene expression datasets [37]. These important approaches to mechanism-based mathematical modeling may also be used to study and predict the effects of perturbations on cell state distributions. They may also be used as computational controls to benchmark analysis tools and potentially to benchmark and compare mathematical models, although using a model to benchmark other models can lead to consistent but incorrect circular reasoning and caution is warranted. Another example is Ferrall-Fairbanks and Papalexi et al, who use mathematical analysis to generate novel quantifications of cell heterogeneity in cancer or immune cell subsets respectively [38, 39]. These methods may be used to map and interpret novel cell states predicted by mathematical models or similarly as a method to interpret model-predicted changes in cell heterogeneity following a perturbation.

Schiebinger et al compute and predict differentiation trajectories in cell development using optimal transport (OT) [40,41]. This approach considers the optimal transport of cells as a mass flowing along differentiation trajectories, and is conceptually the most similar to our approach. As presented, Schiebinger et al do not use the OT framework to examine perturbations of cell states or genes along the differentiation trajectory, although this is possible with an OT model. Setty et al present a method to compute cell fate probabilities [42], which may also be achieved by inferring cell state-transition dynamics with lineage trees [43]. Fischer et al have demonstrated a method for inferring population dynamics from single-cell sequencing data [44], where the model equation is identical to our graph based model developed in [12]. Jiang et al develops a dynamic inference approach to derive a Fokker-Planck type PDE on a graph considering an energy landscape and optimal transport [45]. Sharma et al use longitudinal sequencing to study drug-induced infidelity in the stem cell hierarchy [46], and Karaayvaz et al show how to use single-cell sequencing to examine drug resistance in breast cancer [47]. These approaches and analysis methods may be used to inform and potentially calibrate mathematical models of cell population dynamics or response to treatment-induced perturbations.

Recently, vector fields derived from RNA velocity [48] have been used to infer potential energy or fitness landscapes for cell state-transitions. These approaches may be used to inform the computational domain for mathematical models that we present here, however, an important limitation of the RNA-velocity approach is extrapolation of the vector field outside of the data range. This underscores the need for hypothesis-based and model-guided approaches to inform the shape of these fields. This limitation also applies to the rapidly growing field of deep learning approaches [49] to analyze single-cell sequencing data, namely, whether the learning algorithm can effectively make predictions to datasets which are not sufficiently similar to those upon which it has been trained. We believe that the future likely involves a merger of mathematical modeling with machine learning, in which mathematical models are used to inform learning approaches and impute sparse data as has been recently shown by Gaw and Rockne et al [50, 51]. Among the recent works that align with this direction, PRESCIENT algorithm aims to learn the underlying differentiation landscape from time-series scRNA-seq data [21]. Moreover, dynamo framework improves RNA velocity using kinetic models to reconstruct continuous vector fields that predict cell fates [52].

### 4.2. Opportunities and limitations of modeling with single-cell sequencing data

There are pros and cons, opportunities and limitations to mathematical modeling with single-cell sequencing data. The advantages and potential opportunities include: a wealth of available data, richness and complexity of each data set, a focus on the cell level, opportunity to study dynamics in hierarchically structured state-based relationships between cells, and an ability to perturb individual cells and/or genes within cells to predict dynamics of state-change at cellular level. The most significant strength of mathematical modeling is the ability to use and generate hypotheses that may not be directly evident from the data; for example, extrapolation of RNA velocity fields beyond the dataset boundaries or to interpret and predict novel cell states which may not otherwise be clearly identified with known canonical cell state markers. Another advantage of our approach is the use of pseudo-time analysis of data collected at a single timepoint to calibrate the models, however, the models can also be calibrated directly to time-sequential single-cell datasets, which we expect to become more commonly available as single-cell sequencing continues to be used as a tool to study cell dynamics.

The disadvantages and limitations include: the potential for misleading or incorrect inference due to poor data quality including drop-outs, small non-representative samples of large heterogeneous populations, batch effects, no physical or micro-environmental context, no direct or physical interactions between cells, and the possibility of model predictions to be sensitive to methods of dimension reduction, graph abstraction, state-space construction, and potentially sequencing platform. Sensitivity of the modeling to experimental and computational methods may be directly studied and mitigated as we have shown in this work, however this remains potentially a significant source of uncertainty and variability in the modeling calibration and predictions. Studying the sensitivity of our modeling framework regarding different noise scenarios and applying noise reduction methods is our future work [53].

In terms of computational cost, the graph model is more efficient since it is a multiple of one-dimensional cost, while the cost of implementing the space model increases exponentially as the dimension of reduced space increases. In our simulation, the computational cost to simulate up to time *t* = 50 with step size Δ*t* = 10^−3^ and O(100^2^) degrees of freedom in one-dimension is around 25 seconds in the graph model with 8 nodes, while it takes around 230 seconds in the continuum model with two dimensions. In short, the continuum model runs approximately 10 times longer than the graph model with 8 nodes in our example. Therefore, the multi-dimensional cell state geometry will be reasonable only when the reduced component can be truncated at two- to three-dimension, unless the numerical method is carefully implemented, and we emphasize that the graph model will be more advantageous in terms of computational cost than the continuum model especially when higher dimensional reduced space is necessary.

### 4.3. Future work and applications

Future applications of this approach is to explore hypothesis in the resolution of single-cell genomics and study altered and novel cell states with genetic and epigenetic alterations in various biological systems and pathogenesis. We look forward to compare the model prediction to sampling/sequencing of perturbed biological system, for instance, to examine scRNA-seq data from leukemic progenitor cells. Moreover, we anticipate to incorporate effects of external perturbations such as therapy in future studies.

There are opportunities for further enhancements in our model in improving the model of cell landscape dynamics to accurately estimate cell transition pathways in the reduced component space, for instance, minimum action paths [6] and bifurcation [7, 54]. The model can be improved by obtaining parameter functions or mappings of biological quantities directly from single-cell sequencing data, for example, more precisely infer the proliferation rate function. Also, developing methodologies to obtain reduced component space that captures desired characteristic of cell states [55] will help us explore our approach for other biological settings where cell states are less clearly characterized. Moreover, we propose to develop quantities, such as index of critical state transitions [54,56], in the phenotype space that could be used to predict forthcoming major alterations in development and diseases. We also expect to be able to infer the potential landscape directly from the RNA velocity vector field [48,52].

## 5. Conclusions

In summary, despite the explosion of computational tools to analyze single-cell sequencing data, there have been relatively few mathematical models developed which utilize this data. Here we begin to explore the possibilities—and limitations—of dynamical modeling with single-cell RNA-seq data. We hope this work paves the way for development of mathematical models to guide the interpretation of these complicated datasets as they begin to be collected after biological perturbations (eg., cancer, treatment, altered developmental processes), sequentially over time, or sampled spatially within biological tissues.

## Figures and Tables

**Table 1.** Comparison of the cell state model on graph versus multi-dimensional space in n dimensions. The computational cost is estimated by denoting M as the number of discretized grid points in one-dimension. We comment that the computational cost of a PDE solver can be up to a third power of the degree of freedom.

	Graph model	Multi-dimensional model
Cell state Interpretation	Comprehensible as intermediate cell states	Difficult to interpret
Cell state Exploration	Limited to graph structure	Freedom to explore novel and unconventional cell states
Computational cost (Degree of freedom)	O(*M*)	O(*M^n^*)

**Figure A3.**
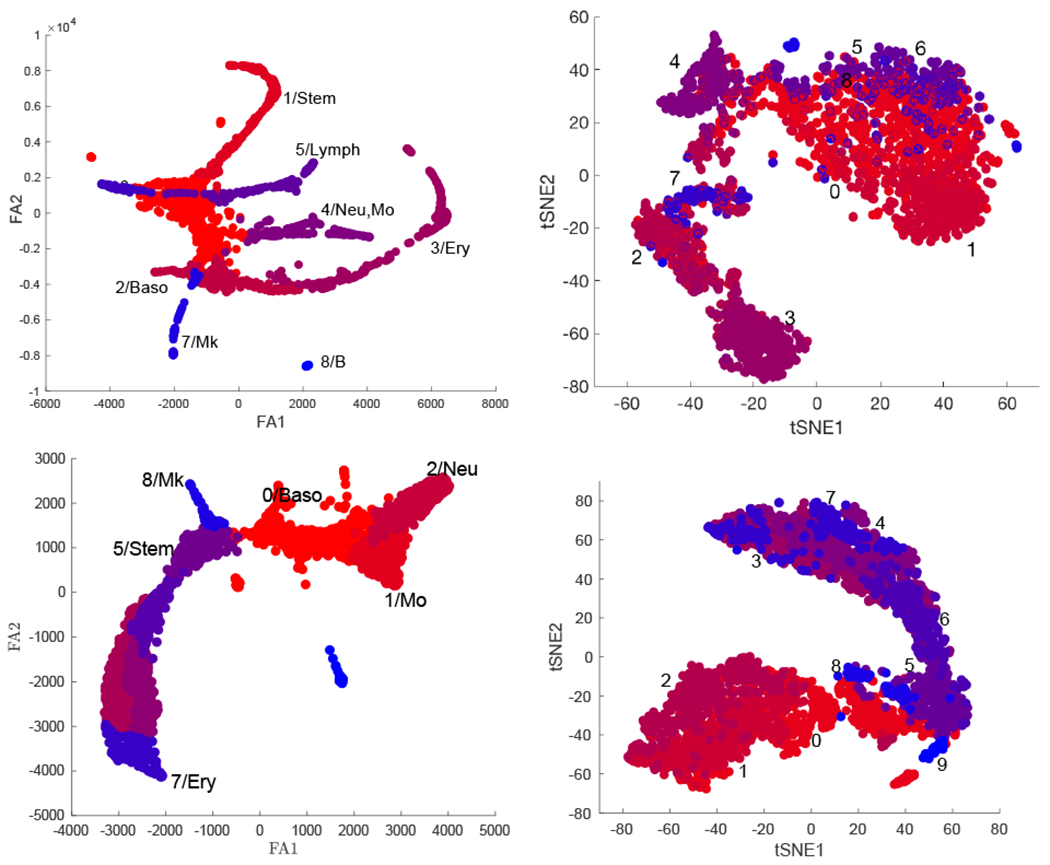
Comparison of dimension reduction methods. Dimension reduction algorithms that focus on preserving local structure are not appropriate to infer global trajectory. Compare the following figures to diffusion component space. They are computed with Nestorowa (top) and Paul (bottom) data and projected on the reduced component space of ForceAtlas2 (FA) [64] and t-stochastic neighbor embedding (tSNE).

**Figure A4.**
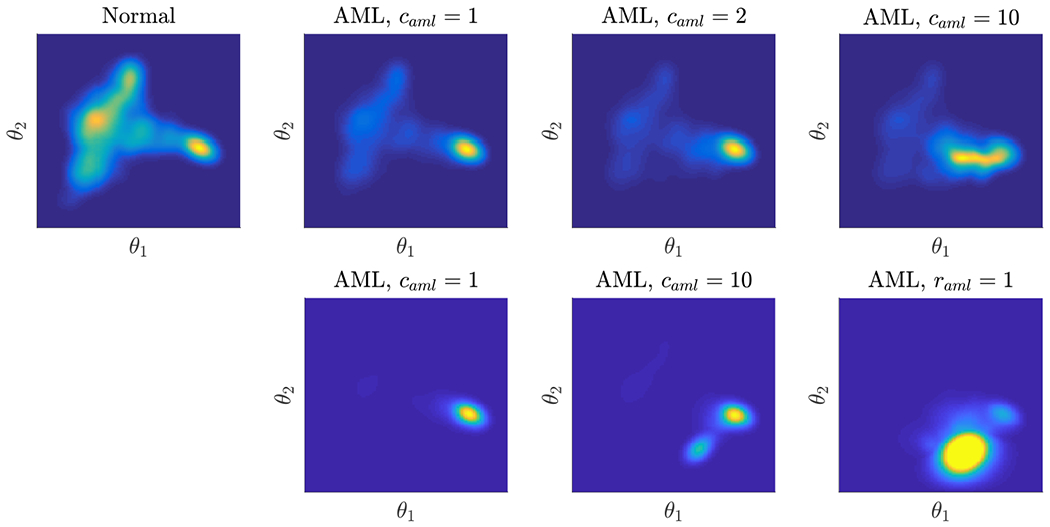
Abnormal cell state transitions during leukemia pathogenesis and progression. The distribution of cell states *u*(*t*, *θ*) show abnormal cell states emerging during leukemic progression after *t* = 10 modeled in the advection term as $V=c_{aml}v_{aml}^{1}$ (top) and $V=v_{1}+c_{aml}v_{aml}^{2}$ (bottom), with various levels of *c*_*aml*_ = 1, 2, 10. Larger magnitude of *c*_*aml*_ results in more disrupted cell landscape.

## Appendix

### A1. Details of the model equation and parameters

The model terms require interpolation of single-cell data to the continuum cell state space. We use clustering to identify cell types and their cell type properties to assign those to each single-cell. The following are the values we take for cluster properties.

By denoting ${\bar{r}}_{i}$ as the assigned proliferation rate of the *i*-th cluster, we compute the intermediate level of proliferation in the graph model by linear interpolation as

(A.1) $$r_{k}\left(x\right)=r_{I\left(i,j\right)}\left(x\right)={\bar{r}}_{i}+\left({\bar{r}}_{j}-{\bar{r}}_{i}\right)x,\;x\in \left[0,1\right],$$

assuming that the overall proliferation of intermediate cell states change gradually. In the multi-dimensional model, we compute the interpolation based on local means as

(A.2) $$r\left(\theta \right)=\frac{1}{\left|I_{\theta }\right|}\underset{i\in I_{\theta }}{\sum }{\bar{r}}^{i},\;I_{\theta }=\left\{i|\left\|\theta^{i}-\theta \right\|<\bar{\theta }\right\},$$

where we take $\bar{\theta }=0.04$. The self-renewal rate functions *a*_*k*_(*x*) and *a*(*θ*) are computed similarly. See Cell proliferation rate *r*(*θ*) and self-renewal rate *a*(*θ*) computed from the single-cell data. The black dots are the rates of data. for *r*(*θ*) and *a*(*θ*) computed for Nestorowa data.

To compute the multi-dimensional function on the continuum space from the single-cell data, we employ the *kernel density method* [18,26], that is a non-parametric way to estimate the density function based on a finite data sample. Using the single-cell samples in the reduced component space, ${\left\{\theta^{i}\right\}}_{i=1}^{N}$, the method approximates the density function as

$$u_{s}\left(\theta \right)=\frac{1}{Nh}\overset{N}{\underset{i=1}{\sum }}K\left(\frac{\left(\theta -\theta^{i}\right)}{h}\right),$$

where *K* is the kernel smoothing function that we take it as a Gaussian function and *h* is the bandwidth. The optimal bandwidth to estimate normal densities can be computed by ${\left(4{\hat{\sigma }}^{5}/3N\right)}^{1/5}$, where $\hat{\sigma }$ is the standard deviation and *N* is the sample size, and the optimal bandwidth for our data is computed as 0.0383 to 0.0456, however in our simulation, we choose a slightly smaller value, *h* = 0.03, to reveal more features of multiple modes. Figure 2(C,D) shows the results computing *u*_*s*_(*θ*) using Nestorowa and Paul data, and A2(a) shows the corresponding **ν**_1_(*θ*).

In the diffusion term, we explore the parameter *ν* so that the phenotypic instability does not dominate the cell maturation. We compute the parameter in the range of $v\leq {\left(L/T_{d}\right)}^{2}/4$, where the distance in the diffusion space is *L* = 1 and the time that HSC differentiates to the progenitors is *T_d_* = 5 ~ 30 (day), that is, *ν* < 0.0027 ~ 0.01, and we consider *ν* = 0.001. Quantifying the local phenotypic instability in the reduced component space, and justifying this term is our future work.

To compute the reduced component space using dimension reduction approaches, we employ *diffusion mapping.* See [15, 62] for the detail of the algorithm. We take the cosine distance, *k*(*x^i^, x^j^*) = 1 − *corr*(*x^i^, x^j^*) for the Nestorowa data and the gaussian distance $k\left(x^{i},x^{j}\right)=\exp\left(-\frac{{\left\|x^{i}-x^{j}\right\|}^{2}}{2\sigma^{2}}\right)$ for Paul data with *σ* = 50. From *L*(*i, j*) = *k*(*x^i^, x^j^*), the diffusion mapping use parameter *α* to tune the influence of density of the data points as

$$L^{\left(\alpha \right)}=D^{-\alpha }LD^{-\alpha },\;M={\left(D^{\left(\alpha \right)}\right)}^{-1}L^{\left(\alpha \right)},$$

where $D^{\left(\alpha \right)}\left(i,j\right)=\sum_{j}L^{\left(\alpha \right)}\left(i,j\right)$, and we choose *α* = 0.5. From the eigen-decomposition of *Mϕ = λϕ* and ordered eigenvalues 1 = *λ*_0_ ≤ *λ*_1_ ≤ *λ*_2_ ≤ ⋯, the corresponding right eigenvectors, *ϕ*_1_, *ϕ*_2_, ⋯ are the diffusion components. We truncate the reduced space at the second diffusion component, where the eigenvalues are *λ*_1_ = 0.1039, *λ*_2_ = 0.0326, *λ*_3_ = 0.0167, *λ*_4_ = 0.0135 for Nestorowa data, and *λ*_1_ = 2.4653e-03, *λ*_2_ = 5.8338e-04, *λ*_3_ = 9.7792e-05, *λ*_4_ = 7.0364e-05 for Paul data. For a comparison of diffusion mapping to two-dimensional reduced component space using other dimension reduction algorithms, see Comparison of dimension reduction methods. Dimension reduction algorithms that focus on preserving local structure are not appropriate to infer global trajectory. Compare the following figures to diffusion component space. They are computed with Nestorowa (top) and Paul (bottom) data and projected on the reduced component space of ForceAtlas2 (FA) [64] and t-stochastic neighbor embedding (tSNE). .

For the pseudotime inference, we use the algorithm developed in [63]. The diffusion distance between two cells are computed as

$$D_{t}^{2}\left(x^{i},x^{j}\right)=\overset{n}{\underset{k=1}{\sum }}\lambda_{k}^{2t}{\left(\phi_{k}^{i}-\phi_{k}^{j}\right)}^{2},$$

and the pseudotime distances are computed based on this distance. We choose three extreme points in each of the three clusters, stem cell, Ery, and Neu cell types, that are the furtherest in the diffusion component space, and infer the lineage between the extreme cells. After computing the pesudotime of each single-cell we compute the local average direction to the neighborhood cells that are in later pesudotime similar as in Eq (A.2). The computed results are shown in Pseudotime dynamics. The homeostasis cell differentiation vector **v**_1_ (a), and the direction of active cell differentiation obtained from diffusion pseudotime analysis (b), and that interpolated at the grid points **v**_2_ (c) are presented. We remark that, **v**_2_ corresponds to the cell differentiation along the edges in the graph model. (b) with the interpolated vector at the grid points, Pseudotime dynamics. The homeostasis cell differentiation vector **v**_1_ (a), and the direction of active cell differentiation obtained from diffusion pseudotime analysis (b), and that interpolated at the grid points **v**_2_ (c) are presented. We remark that, **v**_2_ corresponds to the cell differentiation along the edges in the graph model. (c).

**Table A1.** Summary of the required parameters. The following table summarizes the parameters in our model terms, *V, R, D, V_k_, R_k_*, and *D_k_*, and their biological meaning with the range. The ranges are found from the literature [19,57–61] experimentally measuring the cell cycle and self-renewal rate of the well known hematopoeisis cell types.

Parameters	Biological meaning	Range	
*r*(*θ*), *r*_*k*_(*x*)	proliferation rate	[0.00215, 1]	[57–60]
*a*(*¸*), *a*_*k*_(*x*)	self-renewal rate	[0.1, 0.8]	[19,61]
***c***(*θ*)	differentiation vector	[0, 1]	estimated
*v*	phenotypic fluctuation	[0, 0.0027]	estimated
$\bar{d}$	apoptosis rate	0.6925	[61]

**Table A2.** Parameter values of proliferation and self-renewal rate. The following values are taken for each single-cell, ${\bar{r}}^{i}$ and ${\bar{a}}^{i}$, based on their clustered cell types [19,57–61], and then used for computing *r*(*θ*), *r*_*k*_(*x*), *a*(*θ*), and *a*_*k*_(*x*).

	hematopoietic stem cells (HSC) ↔ progenitor cells (HPC)			

cell type	HSC	MPP, LMPP, CMP	MEP, GMP	Neu/Mo, Ery
proliferation	0.01125	0.05658	0.1612	0.6931
	(8.8 weeks)	(12.25 days)	(4.3 days)	(1 day)

self-renewal	0.77	0.7689	0.7359	0.66

**Table A3.** Gene alterations in Leukemic stem cells. From the genes that are reported in [35, 36], we find all the gene that are in Nestorowa and Paul data. The following table is the genes and their altered magnitude. See [35] Extended Data Table 1 for the 17 genes and [36] Supplemental Table S4 for approximately 80 genes.

Up-regulated Gene	log2-fold change	Down-regulated Gene	log2-fold change
CD34	2.1500	LGALS3	−3.4901
LAPTM4B	1.8000	CYBB	−2.9546
MMRN1	1.3600	CD36	−2.7661
SOCS2	1.2400	ANXA5	−2.6349
CDK6	1.2300	LY86	−2.5564
CPXM1	1.2000	IRF8	−2.4982
EMP1	1.0100	SAMHD1	−2.4580
GPR56	2.7004	GRN	−2.3659
GATA2	1.8875	RNASE6	−2.3585
LPIN1	1.6323	FCER1G	−2.2934
MZB1	1.4854	S100A9	−2.2447
ZSCAN18	1.3219	TLR4	−2.1078
GUCY1A3	1.2630	FCGRT	−2.1016
SPNS2	1.2016	S100A8	−2.0116
PTK7	1.2016	CLEC12A	−1.8730
ABCC1	1.1375	MNDA	−1.8417
SYTL1	1.0704	IL13RA1	−1.7515
MAGED1	1.0704	SGK1	−1.7418
ARHGAP25	1.0704		
SLA2	1.0000		
